# Poly(Propylene Imine) Dendrimers and Amoxicillin as Dual-Action Antibacterial Agents

**DOI:** 10.3390/molecules201019330

**Published:** 2015-10-23

**Authors:** Natalia Wrońska, Aleksandra Felczak, Katarzyna Zawadzka, Martyna Poszepczyńska, Sylwia Różalska, Maria Bryszewska, Dietmar Appelhans, Katarzyna Lisowska

**Affiliations:** 1 Department of Industrial Microbiology and Biotechnology, Faculty of Biology and Environmental Protection, University of Lodz, 12/16 Banacha Street, 90-237 Lodz, Poland; E-Mails: nwronska@biol.uni.lodz.pl (N.W.); afrycie@biol.uni.lodz.pl (A.F.); zawadzka@biol.uni.lodz.pl (K.Z.); n37@o2.pl (M.P.); rozalska@biol.uni.lodz.pl (S.R.); 2 Department of General Biophysics, Faculty of Biology and Environmental Protection, University of Lodz, 141/143, Pomorska Street, 90-236 Lodz, Poland; E-Mail: marbrys@biol.uni.lodz.pl; 3 Leibniz Institute of Polymer Research Dresden, Hohe Street 6, 01069 Dresden, Germany; E-Mail: applhans@ipfdd.de

**Keywords:** poly(propylene imine) dendrimers, antibacterial activity, amoxicillin

## Abstract

Besides acting as antimicrobial compounds, dendrimers can be considered as agents that improve the therapeutic effectiveness of existing antibiotics. In this work we present a new approach to using amoxicillin (AMX) against reference strains of common Gram-negative pathogens, alone and in combination with poly(propylene imine) (PPI) dendrimers, or derivatives thereof, in which 100% of the available hydrogen atoms are substituted with maltose (PPI 100%malG3). The concentrations of dendrimers used remained in the range non-toxic to eukaryotic cells. The results indicate that PPI dendrimers significantly enhance the antibacterial effect of amoxicillin alone, allowing antibiotic doses to be reduced. It is important to reduce doses of amoxicillin because its widespread use in medicine could lead to the development of bacterial resistance and environmental pollution. This is the first report on the combined antibacterial activity of PPI surface-modified maltose dendrimers and amoxicillin.

## 1. Introduction

The widespread use of antibiotics has led to an increase in the number of strains resistant to major antibacterial pharmaceuticals. Therefore, bacterial infections are still leading causes of hospitalization and mortality [1]. In the last few decades, many studies have focused on developing novel and effective antimicrobial agents. The use of nanotechnology in the design and delivery of antimicrobial drugs and control of cross-infections, especially in overcoming resistant pathogens, has been examined as an alternative to current antibiotics-based approaches [2,3]. Bioactive nanomaterials which have many biological applications have been widely described in literature [4,5].

Dendrimers have proved potent in a range of pharmacological applications such as drug and gene delivery and as contrast agents. They are highly branched polymers with well-defined dimensional structures and topological architectures. Dendrimers can be modified with various functional end groups and have found diverse applications in medicine [6,7,8,9,10].

Literature data concerning biomedical applications of these polymers have often focused on the polyamidoamine (PAMAM) dendrimers, which were commercialized very early after their discovery in the 1980s [11,12]. However, knowledge about the applicability of poly(propylene imine) (PPI) dendrimers as antibacterial agents is scant [13,14,15]. Dendrimers can be considered not only as potential antimicrobial compounds but also as agents that enhance the antibacterial or antifungal activities of known drugs.

One of the most commonly-used antibiotics is amoxicillin (AMX) [16]. It is a popular beta-lactam antibiotic, a drug of first choice for treating many types of bacterial infection [17]. It binds to PBP-1A (penicillin-binding protein 1A) inside the bacterial cell wall and, as a consequence, inhibits the third and final stage of cell wall synthesis. Unfortunately, many bacterial strains are resistant to beta-lactam antibiotics, which is problematic for medicine [12]. Gram-negative bacteria most commonly become resistant to beta-lactam antibiotics by producing beta-lactamase [18]. Other mechanisms of resistance include alterations in penicillin-binding proteins (PBPs), impaired permeation of the antibiotic into the bacterial cell, or combinations of those strategies [19]. The correlated effect of PPI dendrimers and antibiotics has seldom been investigated, in contrast to PAMAM dendrimers [20,21]. In this paper we describe the antibacterial activity of amoxicillin in combination with PPI dendrimers. 

## 2. Results and Discussion

### 2.1. Combined Antimicrobial Activity of Amoxicillin and PPI Dendrimers Modified/Unmodified

The widespread use of beta-lactam antibiotics in human and veterinary medicine has resulted in environmental pollution. It has been documented that 30%–90% of ingested antibiotics are excreted unchanged [22]. Beta-lactam antibiotics are among the most prevalent pharmaceutical contaminants currently being detected in aquatic environments [23]. Any trace of the antibiotic in the water can affect aquatic ecosystems adversely; because of this, it is necessary to reduce the use of amoxicillin. This effect can be achieved by simultaneous administration of AMX and macromolecules such as dendrimers.

The main aim of the research was to investigate whether co-administration of PPI dendrimers and AMX enhanced the antimicrobial effect of the latter. The main problem with the biomedical application of nanoparticles is their toxicity. Recently, a series of studies has demonstrated that cationic dendrimers in high concentrations can be toxic to eukaryotic cells. In order to reduce the toxicity of PPI dendrimers, different chemical modifications of their surfaces have been established to neutralize the cationic charge. The replacement of some or all the cationic groups on fourth generation PPI dendrimer surfaces by maltose and maltotriose significantly reduced their toxicity [24]. Dense-shell glycodendrimers, described as amphiphilic macromolecules with a cationic core and a neutral surface charge (PPI dendrimers in which 100% of the available hydrogen atoms are substituted with maltose, PPI 100%malG3) have been found to be non-toxic to the cell lines studied [13,21,25].

**Figure 1:**
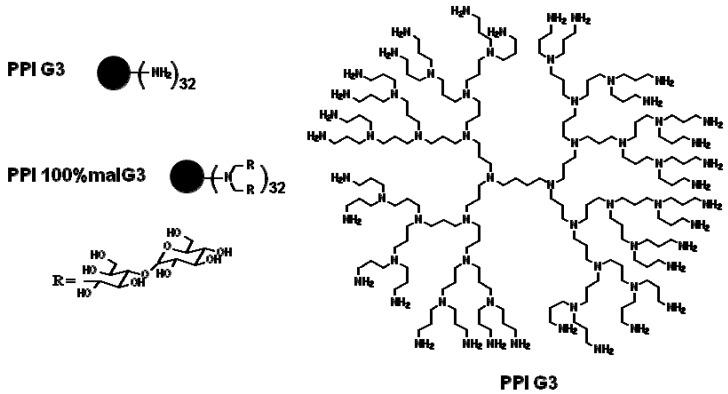
Structures of PPI G3 dendrimers unmodified (PPI) and modified with maltose in 100% (PPI 100%malG3).

We used two types of dendrimers: unmodified poly(propylene imine) dendrimers, and their derivatives in which maltose was substituted for 100% of the available hydrogen atoms, in concentrations that are non-toxic to eukaryotic cells (Figure 1).

The antimicrobial activities of the tested compounds were measured on two commonly-used Gram-negative pathogens: *Escherichia coli* ATCC 25922 and *Pseudomonas aeruginosa* ATCC 15442. Infections caused by Gram-negative strains are serious problems in health care [26]. Beyond the enzymatic mechanisms responsible for the resistance of Gram-negative bacteria, there is also a factor related to outer membrane permeability, which limits the access of drugs to their intracellular targets. Because of the unique features of these bacteria, infections are very difficult to treat. A correlated action of PPI dendrimers (modified, unmodified) with AMX against these two most common human pathogens has not been reported.

**Figure 2:**
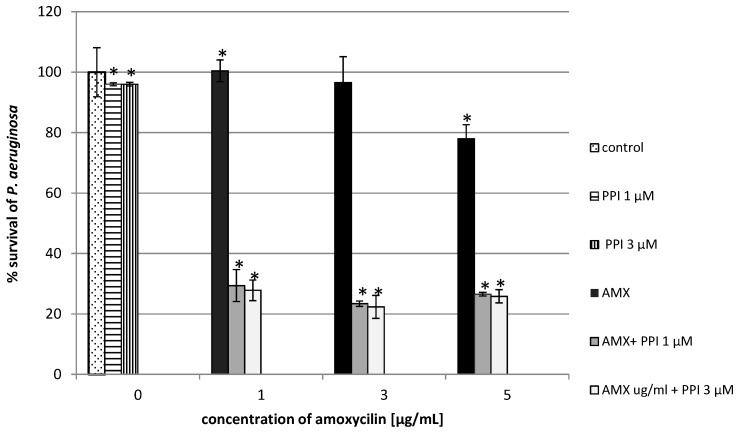
Growth of *P. aeruginosa* after 24 h incubation with unmodified poly(propylene imine) dendrimers (PPI) or/and amoxicillin (AMX). Each bar represents and the mean and SD of *n* ≥ 3 wells from three independent experiments. The comparison was made using one-way analysis of Student’s *t*-test. * *p* < 0.05 *vs.* control group.

**Figure 3:**
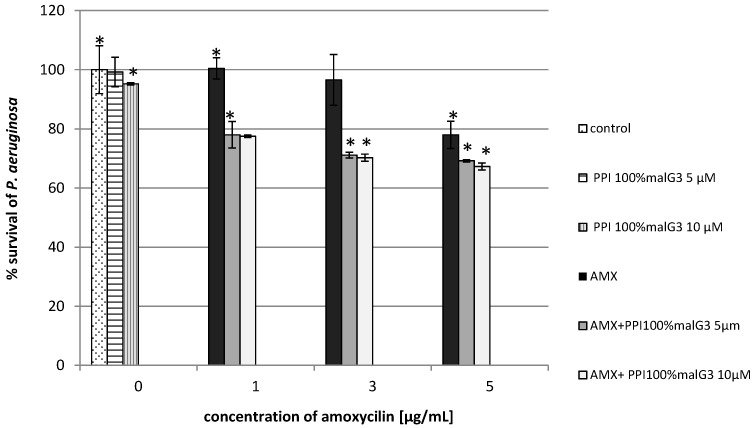
Growth of *P. aeruginosa* after 24 h incubation with poly(propylene imine) dendrimers with a surface modified by attaching maltose in 100% (PPI 100%malG3 or/and amoxicillin. Each bar represents and the mean and SD of *n* ≥ 3 wells from three independent experiments. The comparison was made using one-way analysis of Student’s *t*-test. * *p* < 0.05 *vs.* control group.

Our results showed that the addition of dendrimers (unmodified and maltose-modified) enhanced the antimicrobial effect of AMX for the microorganisms tested. Co-administration of PPI and amoxicillin considerably reduced the growth of *Pseudomonas aeruginosa*. The addition of unmodified PPI dendrimers and amoxicillin limited bacterial growth by 75% at the lowest dose of the dendrimers and the antibiotic, whereas for the dendrimer or the antibiotic applied alone, growth was inhibited by only 5% or 3%, respectively (Figure 2). In cultures containing the maltose-modified dendrimers and the tested antibiotic, the reduction of bacterial growth was 30% higher in comparison to control (Figure 3). In the case of *P. aeruginosa*, the antibiotic (at the lowest concentration) or the dendrimers (modified, unmodified) alone did not inhibit bacterial growth. Only the combination of these compounds gave satisfactory results.

**Figure 4:**
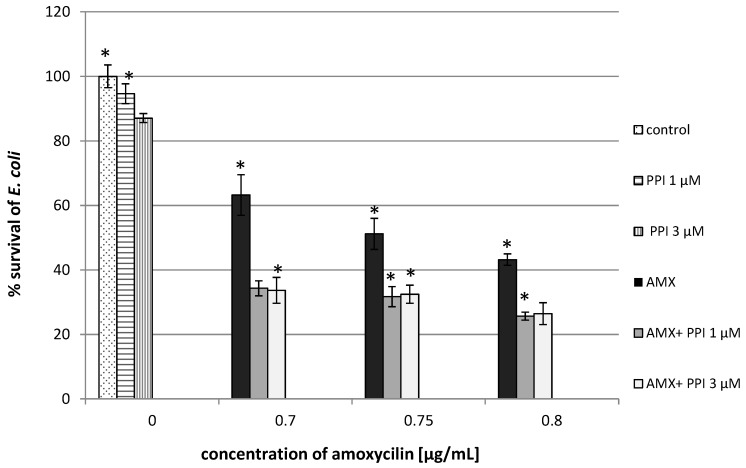
Growth of *E. coli* after 24 h incubation with unmodified poly(propylene imine) dendrimers (PPI) or/and amoxicillin. Each bar represents and the mean and SD of *n* ≥ 3 wells from three independent experiments. The comparison was made using one-way analysis of Student’s *t*-test. * *p* < 0.05 *vs.* control group.

**Figure 5:**
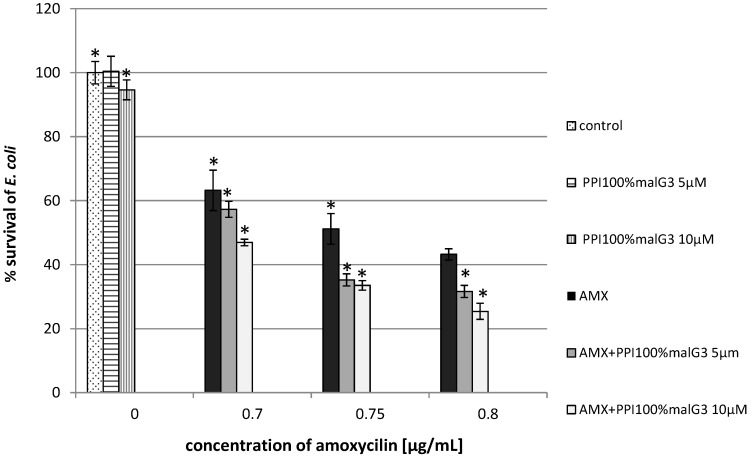
Growth of *E. coli* after 24 h of incubation with poly(propylene imine) dendrimers with a surface modified by attaching maltose in 100% (PPI 100%malG3) or/and amoxicillin. Each bar represents and the mean and SD of *n* ≥ 3 wells from three independent experiments. The comparison was made using one-way analysis of Student’s *t*-test. * *p* < 0.05 *vs.* control group.

Promising results were also obtained for *E. coli.* The addition of unmodified PPI dendrimers (1 µM) enhanced the antibacterial activity of the beta-lactam antibiotic at the lowest concentration by 50% (Figure 4). Similar results were obtained with maltose-modified dendrimers (PPI 100%malG3 + AMX). At concentrations of 0.75 µg/mL antibiotic and 10 µM dendrimers, the bacterial growth was inhibited by 70%, whereas for the antibiotic applied alone, growth was limited by 47% (Figure 5).

This is in agreement with our previous results showing that PPI dendrimers enhanced the antibacterial activity of nadifloxacin against Gram-negative organisms [14]. These pathogens exhibited relatively high tolerance to the dendrimers applied alone: PPI dendrimers inhibited the growth of these bacteria by only 10%–20%, even when high concentrations (100 µM) were used. These macromolecules exhibited their highest antimicrobial activity against the Gram-positive bacterium *S. aureus* [13]. Compared to unmodified PPI dendrimers, PPI glycodendrimers (PPI 100%malG3) had much weaker antibacterial activities against Gram-positive bacteria, and Gram-negative bacteria were resistant to them [13,14].

A novel antimicrobial agent should be non-toxic to eukaryotic cells and possess potent antimicrobial activity. On the basis of our previous results we concluded that modified dendrimers (PPI 100%malG3) at the concentrations 1–100 µM were almost non-toxic to eukaryotic cells. Similar results were obtained for PPI dendrimers, but only at very low concentrations (up to 3 µM). Cytotoxicity of analyzed dendrimers was studied in relation to the Chinese hamster fibroblast cell line (B14), a human liver hepatocellular carcinoma cell line (HepG2), a mouse neuroblastoma cell line (N2a) and rat liver cell line (BRL-3A) [13,14,15]. Published data indicate the influence of dendrimers surface modification on their cytotoxicity [27,28,29]. Janaszewska *et al.* showed that modification of a dendrimers surface by maltotriose clearly reduced toxicity toward Chinese hamster ovary (CHO) and human carcinoma (SKON3) cells [30]. Other studies have shown that oligosaccharide-modified PPI dendrimers exhibit good solubility under physiological conditions [21,31]. PAMAM dendrimers are promising drug delivery carriers because of their amine-terminated chains, but their cytotoxicity has limited their clinical use [32]. Most studies to date have focused on PAMAM dendrimers surface modification. Wang *et al.* demonstrated that hydroxyl-terminated and amine-terminated PAMAM dendrimers both inhibited bacterial growth, but amine-terminated compounds were cytotoxic at low concentrations (above 10 µg/mL), whereas hydroxyl-terminated ones were non-cytotoxic up to 1 mg/mL [33]. It was found that the hydrophobicity of the PAMAM dendrimer surface group significantly influenced the association of the dendrimer with bacteria, the extent of bacterial membrane disruption, and the cytotoxicity to mammalian cells [34].

Our results showed that PPI dendrimers and PPI glycodendrimers, where maltose was substituted for 100% of the available hydrogen atoms strengthened the antibacterial activity of AMX, allowing the antibiotic dose to be reduced. This is the first attempt to use neutral PPI glycodendrimers with AMX as dual-action antibacterial agents.

### 2.2. Permeability of Bacterial Cell Membranes

In order to confirm that bacterial cell membranes were disrupted in the presence of PPI dendrimers and AMX, we treated selected samples with propidium iodide (PI). This dye is commonly used for discriminating dead from living cells because it can enter only damaged cells with permeable membranes [35].

**Figure 6:**
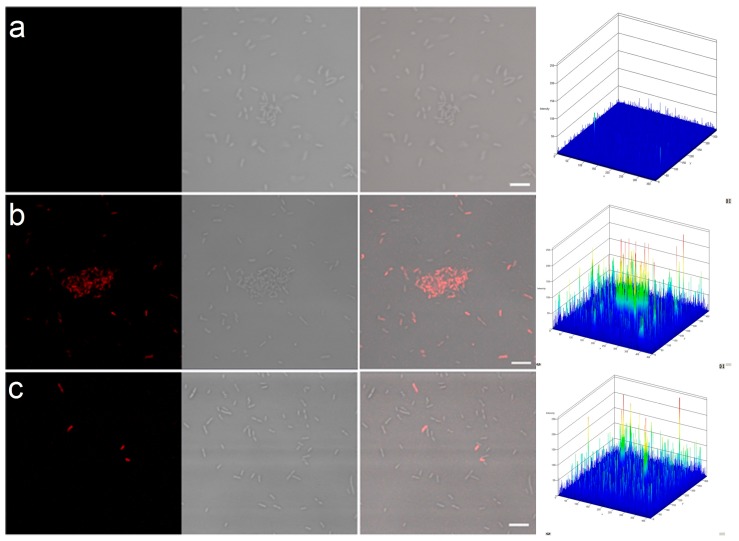
Permeability of cell membranes of *P. aeruginosa* after treatment with PPI dendrimers.

The results indicate that PPI G3 or PPI 100%malG3 dendrimers with AMX exert detrimental effects on bacteria. After *P. aeruginosa* was treated with these compounds, cell membrane permeability to PI increased (Figure 6; Table 1). The highest permeability was noted for PPI dendrimers and AMX (61.88%). The difference in the susceptibilities of Gram-negative bacteria to the tested PPI dendrimers could be attributed to various surface charges and the mass of the macromolecules. PPI dendrimers penetrate through the cell wall more easily than glycodendrimers. Chen *et al.* showed that bacterial membranes have lower permeabilities of the larger dendrimer analogues [36].

(**a**) control; (**b**) PPI 1 µM with AMX 1 µg/mL; (**c**) PPI 100%malG3 5µM with AMX 1 µg/mL. For all photomicrographs the left row shows red fluorescence (propidium iodide, PI, staining) and the right shows Nomarski differential interference contrast.

**Table 1:** PI permeability (%) of cell membranes of *P. aeruginosa* after treatment with PPI dendrimers.

Sample	PI Permeability (%)
control	0.29 ± 0.05
PPI	4.78 ± 2.45
AMX	2.40 ± 0.36
PPI + AMX	61.88 ± 6.01
PPI 100%malG3	2.10 ± 0.19
PPI 100%malG3 + AMX	13.50 ± 3.63

**Figure 7:**
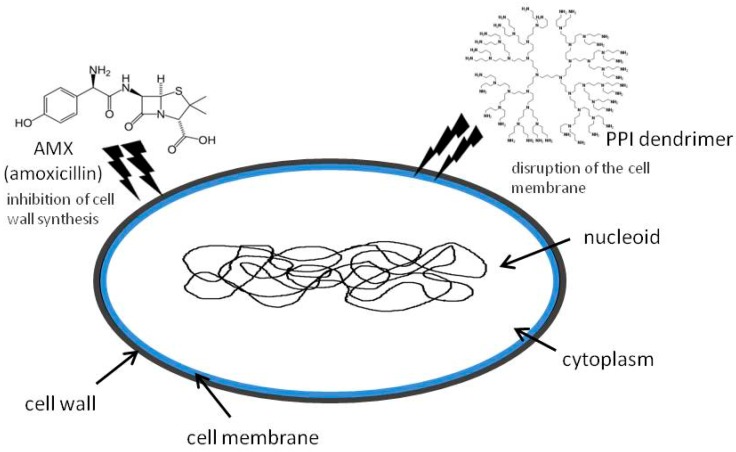
Mechanism of antibacterial activity of PPI dendrimers and amoxicillin.

Destroying the cell membranes of microorganisms directly, or disrupting multivalent binding interactions between microorganisms and host cells, are the primary mechanisms of antimicrobial action by dendrimers [37]. Cationic dendrimers act via initial electrostatic attraction to the negatively charged bacterium followed by membrane and peptidoglycan disruption [38]. Amoxicillin inhibits bacterial cell wall synthesis so it facilitates contact of the dendrimer with the cell membrane (Figure 7). This effect was also observed with glycodendrimers, which did not show such activity even when combined with fluoroquinolone antibiotics. However, those antibiotics have a different mechanism of action, inhibiting two enzymes (DNA gyrase and topoisomerase IV) required for bacterial multiplication [39].

The dual action of dendrimers and β-lactam antibiotics might not be synergistic, as described by Yang and Lopina for the covalent surface conjugation of penicillin V to G-2,5 and G-3 PEG-PAMAM polymers against *S. aureus* [38]. On the other hand, Kannan *et al.* demonstrated that G4 PAMAM dendrimers encapsulating amoxicillin have the potential for enriched antimicrobial activity [39]. PAMAM dendrimers are often used to enhance the pharmacological activities of antimicrobial drugs, as reported for erythromycin and tobramycin [40]. In recent years, the appearance of resistant strains of bacteria has been accompanied by the dearth of new antibiotics, especially those against Gram-negative bacteria [41]. As a consequence, strategies to preserve the activities of existing antimicrobial drugs are urgently needed [42]. In the present work, it was shown that the PPI dendrimers or glycodendrimers have the potential to enhance antibacterial activity against Gram-negative bacteria.

## 3. Experimental Section

### 3.1. Reagents

Unmodified poly(propylene imine) (PPI G3, MM 3514 g/mol) dendrimers were purchased from SyMO-Chem (Eindhoven, The Netherlands). The synthesis of a maltose-modified 3rd generation PPI dendrimer (PPI 100%malG3, MM 24397) was described by Klajnert *et al.* [21]. The degree of maltose substitution in PPI 100%malG3 was determined using ^1^H-NMR [21]. Amoxicillin was purchased from Sigma-Aldrich (Darmstadt, Germany).

### 3.2. Determination of Antimicrobial Activity

The antimicrobial activities of the dendrimers and the antibiotic were examined using a modified broth microdilution method, according to the standards defined by the National Committee for Clinical Laboratory Standards (NCCLS M07-A8). The analysis involved incubating *P. aeruginosa* (ATCC 15442) and *E. coli* (ATCC-25922) with serial dilutions of the compounds on microtiter plates and measuring cell density (OD) spectrometrically at 620 nm. The antimicrobial activity of the dendrimers was determined at 1 and 3 μM for unmodified PPI dendrimers and 5 and 10 μM for maltose-modified ones. The germicidal activity of amoxicillin was measured at different concentrations against the two bacterial strains at 0.7–5.0 µg/mL (see Results). The data were compared to three independent controls: (i) bacteria incubated in the medium; (ii) bacteria incubated with dendrimers; and (iii) bacteria incubated with antibiotics.

### 3.3. Permeability of Bacterial Cell Membranes

Bacterial suspensions obtained after incubation with dendrimers and/or AMX were washed twice with phosphate buffered saline (0.1 M, pH = 7.4) and incubated with 3 µM propidium iodide in the dark for 15 min at room temperature. After the incubation, the bacterial cells were washed twice with PBS and 10 µL aliquots of the suspensions were mounted on microscope slides.

### 3.4. Confocal Microscopy

Images were obtained using a Confocal Laser Scanning Microscope (LSM510 Meta, Zeiss, Oberkochen, Germany) combined with an Axiovert 200M (Zeiss) inverted fluorescence microscope equipped with a Plan-Apochromat objective (63x/1.25 Oil). All settings were held constant throughout all experiments. Propidium iodide fluorescence was measured using the He-Ne laser (543 nm) and an LP filter set (560–615 nm). The Nomarski DIC sections were examined at the excitation wavelength (488 nm) of an argon laser. All figure panels in this article represent typical results.

### 3.5. Statistics

Experiments were carried out at least in triplicate unless otherwise stated. All values were expressed as means ± standard deviation (SD). Statistical significance was established by one-way analysis of Student’s *t*-test with *p* < 0.05 as the threshold.

## 4. Conclusions

It can be concluded that the combined action of PPI dendrimers or glycodendrimers with amoxicillin enhances the antibacterial effect of the antibiotic alone. The results indicate the possibility of reducing the doses of antibiotic applied. This is of great importance owing to the rise of microbial drug resistance and environmental pollution caused by the widespread use of B-lactam antibiotics. This is the first report of the possibility of using the simultaneous action of PPI dendrimers and B-lactam antibiotics against Gram-negative bacteria. The dendrimer concentrations used were in the range that is considered to be harmless to eukaryotic cells.
